# Study of the Antibacterial Efficacy of Bainiku-Ekisu against Pathogens

**DOI:** 10.1155/2014/460395

**Published:** 2014-10-28

**Authors:** Deng-Jye Yang, Hsin-Yi Chen, Shih-Chuan Liu

**Affiliations:** ^1^School of Health Diet and Industry Management, Chung Shan Medical University, 110, Sec. 1, Jianguo North Road, Taichung 402, Taiwan; ^2^School of Nutrition, Chung Shan Medical University, 110, Sec. 1, Jianguo North Road, Taichung 402, Taiwan; ^3^Department of Nutrition, Chung Shan Medical University Hospital, 110, Sec. 1, Jianguo North Road, Taichung 402, Taiwan

## Abstract

The research was undertaken to determine the bacteriostatic effects of the concentrate of Japanese apricot juice (bainiku-ekisu), which is a popular health food in Taiwan and Japan, on *Enterococcus faecalis* ATCC 29212, *Staphylococcus aureus* ATCC 25923, and *Escherichia coli* ATCC 25922. The results show that *E. faecalis*, *S. aureus*, and *E. coli* could be killed or inhibited by bainiku-ekisu at concentrations between 1.0 and 10.0 mg/mL. The minimum inhibitory concentration (MIC) was 1 mg/mL for all strains, and the minimum bactericidal concentrations (MBCs) were 5, 2.5, and 2.5 mg/mL for *E. faecalis*, *S. aureus*, and *E. coli*, respectively. Using the growth rate to calculate the MICs and MBCs, the MICs were 1.55, 1.43, and 0.97 mg/mL, and the MBCs were 2.59, 2.63, and 2.25 mg/mL for *E. faecalis*, *S. aureus*, and *E. coli*, respectively. According to the *D* values, *E. faecalis* and *S. aureus* exhibited lower resistance than * E. coli* at lower bainiku-ekisu concentrations (1.0 and 2.5 mg/mL), and the resistance of these two pathogens was better than that of *E. coli* at higher bainiku-ekisu concentrations (5.0 and 10.0 mg/mL). The *Z* values of the* E. faecalis*,* S. aureus*, and* E. coli* strains were 3.47, 4.93, and 11.62 mg/mL, respectively.

## 1. Introduction

*Enterococcus faecalis*,* Staphylococcus aureus,* and* Escherichia coli* have been recognized as important pathogens that cause human disease and regularly infect hospitalized patients.* S. aureus* and* E. coli* are also common causes of food poisoning. Antibiotics are the major tools to treat bacterial infections and cure patients. However, bacteria are highly adaptable and can develop resistance to antibiotics, yielding resistant pathogens such as methicillin-resistant* S. aureus* (MRSA) [1]. These antibiotic-resistant bacteria pose significant dangers to hospitalized patients because infections with resistant bacteria are difficult to treat with antibiotics. Therefore, it is important to reduce antibiotic consumption to eliminate the development of antibiotic-resistant bacteria.

In clinical settings, antibacterial effects are usually quantified as the minimum inhibitory concentration (MIC) and the minimum bactericidal concentration (MBC) [2]. These methods are easy ways to determine the curative antibiotic dose; however, the dose range is wide. The use of more antibiotics might produce antibiotic-resistant bacteria. The decimal reduction time (*D* value) and the resistance coefficient (*Z* value) are widely used in thermal death time (TDT) data analysis to determine the heat resistance of the bacteria [3]. The difference between the* D* and* Z* values is dependent on concentration and temperature. The* D* and* Z* values can be used to describe the drug resistance of bacteria, and these data can provide a more accurate estimate of the curative dose.

Bainiku-ekisu is the concentrate of Japanese apricot juice. It has a pH of approximately 2.85, and its major components are citric acid and malic acid (40–53%) [4]. Bainiku-ekisu is a popular product in Taiwan and Japan because consumers believe that it has many types of physiological activities, such as antioxidant activity [5], the ability to improve blood fluidity [6], anti-cancer activity [7] and antibacterial activity [8, 9]. Fujita et al. [8] demonstrated that bainiku-ekisu exhibits strong bacteriostatic effects at a concentration less than 0.156% (w/w) against four* Helicobacter pylori* stains and at a concentration less than 0.313% against six* H. pylori* strains. When these ten* H. pylori* strains were mixed with 0.3% and 0.9% bainiku-ekisu, respectively, strong bacteriostatic effects were observed after 5 minutes. Nakajima et al. [9] also demonstrated that the urea breath test (UBT) values of* H. pylori*-positive patients were decreased slightly when the patients were treated with 1% bainiku-ekisu solution for 2 weeks.

According to these studies, bainiku-ekisu exhibits strong bacteriosterile effects; therefore, this product could be used to treat and prevent pathogenic bacterial infections and could reduce antibiotic consumptions. The objectives of this research were to assess the antibacterial effects of bainiku-ekisu on* E. faecalis*,* S. aureus* and,* E. coli* and to measure the minimum inhibitory concentration (MIC), minimum bactericidal concentration (MBC), decimal reduction time (*D* value) and resistance coefficient (*Z* value).

## 2. Materials and Methods

### 2.1. Preparation of Bainiku-Ekisu

Semi-ripe Japanese apricot fruits were harvested from trees and purchased from Hsinyi Hsiang, Nantou Prefecture, located in the middle part of Taiwan, during the spring of 2006. The pits were removed, and the fruit was minced. The minced fruit was filtered through cheese cloth, and the juice was boiled at temperature around 100°C for 8 hours to produce the bainiku-ekisu concentration. Before analysis, bainiku-ekisu was reconstituted to the fresh juice with de-ionized water. In the antibacterial assays, the bainiku-ekisu was diluted with de-ionized water to concentrations of 0.5, 1.0, 2.5, 5 and 10 mg/mL.

### 2.2. Determination of the Characteristics of Fresh Japanese Apricot Juice and Bainiku-Ekisu

All solvents were purchased from Merck Co. (Darmstadt, Germany), and all chemicals were purchased from Sigma Chemical Co. (St. Louis, MO, USA). The fresh Japanese apricot juice and the bainiku-ekisu were analyzed to determine the pH, °Brix, total acidity, total phenolic content and total flavonoid content. The pH values of samples were determined with a pH meter (Model SP-71, Suntex Inc., Taiwan). The °Brix was measured with a hand-held refractometer (ATAGO, Japan). The total acidity of the samples was measured according to the AOAC [10]. The total concentration of reducing sugars was determined using the dinitrosalicylic acid (DNS) method [11].

The total phenolic content was determined using the method in the report of Julkunen-Tiitto [12]. A 50 *μ*L aliquot of sample or standard solution was mixed with 1 mL of de-ionized water and 0.5 mL of Folin-Ciocalteu's phenol reagent. The mixture was then added to 2.5 mL of 20% Na_2_CO_3_ solution and incubated at ambient temperature in the dark for 20 min. The absorbance of the mixture was measured at 735 nm (Spectro UV-Vis Auto Spectrophotometer, Labomed Inc., Culver City, CA, USA). A standard curve was constructed using gallic acid, and the total phenolic content was expressed as mg gallic acid equivalents (GAE)/g sample.

The total flavonoid contents in samples were determined according to the method of Zhishen et al. [13] with some modifications. An aliquot (250 *μ*L) of the sample or a standard solution was mixed with de-ionized water (1.25 mL) and 5% NaNO_2_ solution (75 *μ*L) for 6 min. To the mixture was added 10% AlCl_3_·H_2_O solution (150 mL) and mixed for 5 min, and then 0.5 mL of 1 M NaOH solution was added. The total volume of the mixture was brought up to 2.5 mL with de-ionized water, and the absorbance at 510 nm was determined. The standard curve was constructed using (+)-catechin, and the total flavonoid content was expressed as mg catechin equivalents (CE)/g sample.

### 2.3. Determination of the Antioxidative Activities of Fresh Japanese Apricot Juice and Bainiku-Ekisu

The antioxidative activities of fresh Japanese apricot juice and bainiku-ekisu were determined using DPPH (2,2-Diphenyl-1-picrylhydrazyl) and ABTS^.+^ radical-scavenging assays, and each of these methods is described below.

#### 2.3.1. DPPH (2,2-Diphenyl-1-picrylhydrazyl) Radical-Scavenging Effect

The DPPH radical-scavenging activity was determined using a modification of the method of Espín et al. [14]. Samples of 20 *μ*L each were dispensed into a 96-well plate, and then 200 *μ*L of 0.2 mM DPPH solution (prepared with methanol) was added to each well. These plates were incubated at 37°C in a dark chamber for 30 min and then put in a microplate reader (Multiskan Spectrum, Thermo, Vantaa, Finland) to measure the absorbance at 517 nm (*A*_517_). The DPPH scavenging activity was evaluated based on the percentage of DPPH radicals scavenged using the following equation:

(1) $$\begin{array}{c} S_{\text{DPPH}}=\frac{\left\{S_{b}-\left(S_{c}-S_{s}\right)\right\}}{S_{b}}\times 100, \end{array}$$

where *S*_DPPH_ is the DPPH radical-scavenging activity expressed as a percentage, *S*_*b*_ is the absorbance of the blank treatment, *S*_*c*_ is the absorbance of the sample, and *S*_*s*_ is the background absorbance of the sample.

#### 2.3.2. ABTS^.+^ Radical-Scavenging Effect

This assay was performed using the assay of Scalzo et al. [15] with some modifications. ABTS solution was prepared by mixing peroxidase, H_2_O_2_ and 2,2-azino-bis-[3-ethylbenzothiazoline-6-sulfonic acid] (ABTS) in de-ionized water to yield a final peroxidase activity of 4.4 U/mL, an H_2_O_2_ concentration 100 mM, and an ABTS concentration 50 *μ*M. This solution was incubated at 30°C in a dark chamber for 1 hour. A 30 *μ*L aliquot of each sample was loaded onto a 96-well plate before dispensing 250 *μ*L of ABTS solution into each well. The plate was then put into the microplate reader to measure the absorbance at 734 nm (*A*_734_) after standing for 3 min. The ABTS^.+^ radical-scavenging activity (*S*_ABTS_) was calculated by the following equation:

(2) $$\begin{array}{c} S_{\text{ABTS}}=\frac{\left[A_{b}-\left(A_{c}-A_{s}\right)\right]}{A_{b}}\times 100, \end{array}$$

where *S*_ABTS_ is ABTS^.+^ radical-scavenging activity expressed as a percentage, *A*_*b*_ is the absorbance of the blank treatment, *A*_*c*_ is the absorbance of the sample, and *A*_*s*_ is the background absorbance of the sample.

### 2.4. Measurement of the Antibacterial Effect

*E. faecalis* ATCC 29212 (Gram-positive),* S. aureus* ATCC 25923 (Gram-positive) and* E. coli *ATCC 25922 (Gram-negative) were used to evaluate the antibacterial effects of bainiku-ekisu. These strains were incubated in Mueller Hinton broth (DIFCO, US) at 37°C. After incubation, the concentration of the bacteria was adjusted to approximately 1–5 × 10^5^ CFU/mL. Different bainiku-ekisu concentrations were added to the incubated cultures. The cultures were then incubated at 37°C and sampled at intervals to count the number of bacteria. A 1 mL aliquot of the sample was inoculated onto a Mueller Hinton agar plate (DIFCO, US), and the plates were incubated in an inverted position at 37°C for 24 hours. After incubation, the number of colonies was determined.

### 2.5. Data Analyses

The antibacterial effects of bainiku-ekisu are discussed in the following sections.

#### 2.5.1. Determination of the Minimum Inhibitory Concentration (MIC) and the Minimum Bactericidal Concentration (MBC)

The determination of MIC and MBC is recommended by the NCCLS [2]. The MIC was determined as the lowest concentration of the tested compound in which the number of bacteria was not significantly different from the number of bacteria in the control after 18–24 hours of incubation at 37°C. The MBC was determined as the lowest concentration of the tested compound in which the number of bacteria was reduced by 99.9% relative to the CFU/mL of the control.

#### 2.5.2. Determination of the Decimal Reduction Time (*D* Value) and the Resistance Coefficient (*Z* Value)

The* D* and* Z* values are widely used in thermal death time (TDT) data analyses to determine the resistance of bacteria. The decimal reduction time (*D* value) is calculated from the linear part of thermal death time curve and is equal to the time required to reduce the number of bacteria by a factor of 10 (i.e., one log count reduction). The* Z* value is calculated from the regression of log (*D*) versus temperature and is equal to change in temperature necessary to reduce the decimal reduction time by a factor of 10. In the study, the* Z* value was calculated from the regression of log (*D*) versus the bainiku-ekisu concentration and was equal to the change in concentration necessary to reduce the decimal reduction time by a factor of 10.

### 2.6. Statistical Analysis

All experiments were carried out in triplicate. The data were subjected to analysis of variance (ANOVA), and Duncan's multiple range tests were used to identify significant differences between means at a significance level of *P* < 0.05.

## 3. Results and Discussion

### 3.1. The Characteristics of Fresh Japanese Apricot Juice and Bainiku-Ekisu

To compare fresh Japanese apricot juice and bainiku-ekisu, bainiku-ekisu was diluted to the concentration of fresh juice using de-ionized water. The characteristics of fresh Japanese apricot juice and bainiku-ekisu are shown in Table 1. The pH levels of fresh juice and bainiku-ekisu were lower than pH 3, indicating that the concentration process was performed under acidic conditions. The °Brix, total acidity and total amount of reducing sugars for bainiku-ekisu were significant lower than those for fresh juice. Those results may due to that the Maillard reaction, the caramelization reaction and mumefural generation might take place during juice concentration at high temperatures [6, 16].

The total phenolic and flavonoid contents of bainiku-ekisu were higher than those of fresh juice (Table 1). During heating at high temperature for a long time, phenolic compounds and flavonoids can be destroyed; however, the phenolic and flavonoid contents were found increased in this study. There are a few articles that reported the total phenolic and flavonoid contents of citrus peels increased during high temperature treatment [17–20]. Several low-molecular-weight phenolic compounds might form in* Citrus unshiu* peels heated at 150°C for 30 min [21]. Que et al. [19] proposed that the phenolic compounds might be formed from the precursors of phenolic molecules via non-enzymatic interconversion between phenolic molecules. The increases in the DPPH and ABTS^.+^ radical-scavenging activities could be due to the higher phenolic and flavonoid contents. Furthermore, the products of the Maillard reaction and the caramelization reaction could exhibit antioxidative activities [16, 22, 23]. The conversion of the EC_50 _values for the DPPH and ABTS^.+^ radical-scavenging activities of bainiku-ekisu into weights of the final product yielded values of 2.61 and 3.33 mg/mL, respectively.

### 3.2. Measurement of the Antibacterial Effects

#### 3.2.1. MIC and MBC

**Figure 1:**
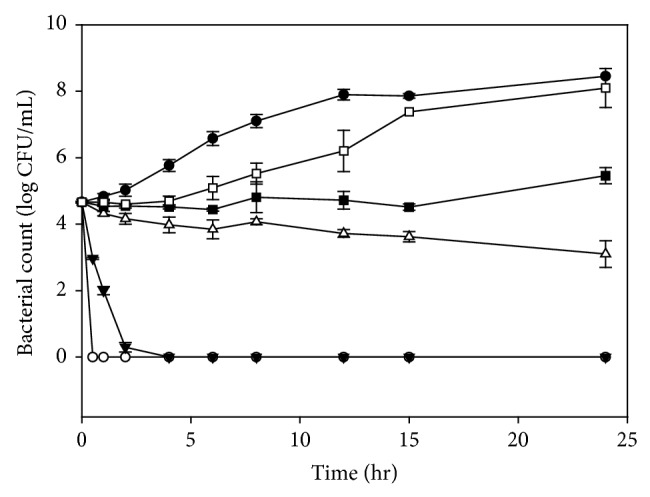
Growth of* Enterococcus faecalis* in the presence of various bainiku-ekisu concentrations. ●, Control (0 mg/mL); □, 0.5 mg/mL; ■, 1 mg/mL; △, 2.5 mg/mL; ▼, 5 mg/mL; ○, 10 mg/mL.

Figure 1 shows the growth patterns of* E. faecalis* when treated with/without different bainiku-ekisu concentrations (0–10 mg/mL). When* E. faecalis* was incubated without bainiku-ekisu, the bacteria counts increased steadily during the 12 hours incubation. The bainiku-ekisu solutions affected the growth patterns of* E. faecalis. *Schoenknecht et al. [24] described 4 types of “killing curves” in clinical research. When 5 and 10 mg/mL bainiku-ekisu solutions were used, the number of bacteria decreased to zero within 4 and 0.5 hours, respectively. The results demonstrate that bainiku-ekisu can inhibit and kill* E. faecalis* (type D curve). The number of bacteria slightly decreased in the presence 2.5 mg/mL bainiku-ekisu. The killing type was similar to type D, but rate of the decrease was slower than that for 5 mg/mL. The killing type of 1 mg/mL bainiku-ekisu was type B, which involves a lag phase increase. The killing type of 0.5 mg/mL bainiku-ekisu was type C, which involves a decrease in the growth rate, with little effect on the lag time. Type A effect (“percentage growth inhibition”) was also observed in this study.

The MIC and MBC of bainiku-ekisu were 1 mg/mL and 5 mg/mL, respectively. Hoellman et al. [25] reported that the MICs of ampicillin and vancomycin for enterococci were 0.3 *μ*g/mL and 4.2 *μ*g/mL, respectively. The amount of bainiku-ekisu used in this study was higher than amounts of antibiotics used in their study, but bainiku-ekisu is a natural product. The antimicrobial activities (MIC and MBC) of* Cyperus rotundus* ethyl acetate extract for* E. faecalis* were 2.5 and 5 mg/mL, respectively [26]. The antimicrobial activities of bainiku-ekisu were better than those of* Cyperus rotundus* ethyl acetate extract.

**Figure 2:**
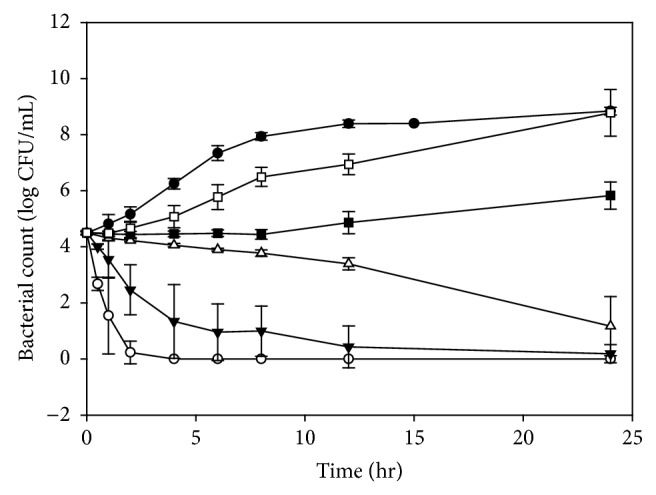
Growth of* Staphylococcus aureus* in the presence of various bainiku-ekisu concentrations. ●, Control (0 mg/mL); □, 0.5 mg/mL; ■, 1 mg/mL; △, 2.5 mg/mL; ▼, 5 mg/mL; ○, 10 mg/mL.

Figure 2 shows that the growth patterns of* S. aureus* when incubated with bainiku-ekisu (0–10 mg/mL). When* S. aureus* was incubated without bainiku-ekisu, the number of bacteria increased steadily during the 12 hours incubation, reaching approximately 10^8^ CFU/mL. The growth patterns of* S. aureus* were affected when bainiku-ekisu solutions were added. When the 5 and 10 mg/mL bainiku-ekisu solutions were added, the number of bacteria decreased to zero within 24 and 4 hours, respectively. The kill rates for* S. aureus* were slower than those for* E. faecalis*. However, the 2.5 mg/mL bainiku-ekisu markedly killed* S. aureus*, resulting in 10^1^ CFU/mL after incubation for 24 hours. This inhibitory activity was better than that against* E. faecalis*. In contrast, the results for the treatment of* S. aureus* with 0.5 and 1 mg/mL bainiku-ekisu were similar to the results for the treatment of* E. faecalis* after a 24 hours incubation. The MIC and MBC of bainiku-ekisu were 1 mg/mL and 2.5 mg/mL, respectively. Similarly, the MIC and MBC of* C. rotundus* ethyl acetate extract for* S. aureus* were 2.5 and 5 mg/mL, respectively [26]. The antimicrobial activities of bainiku-ekisu against* S. aureus* were significant better than the effect of* Cyperus rotundus* ethyl acetate extract.

**Figure 3:**
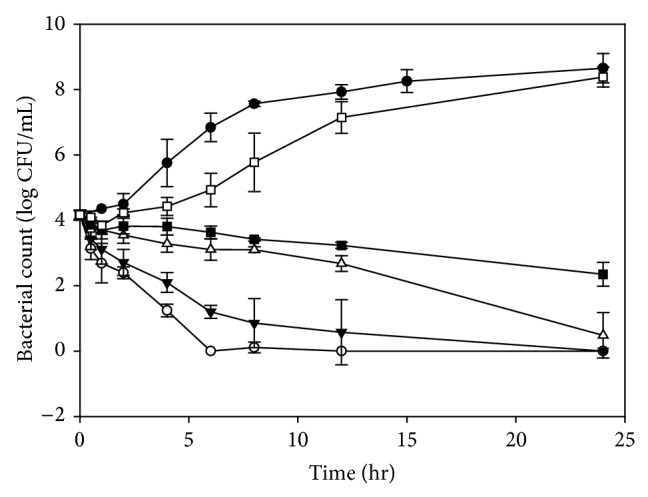
Growth of* Escherichia coli *in the presence of various bainiku-ekisu concentrations. ●, Control (0 mg/mL); □, 0.5 mg/mL; ■, 1 mg/mL; △, 2.5 mg/mL; ▼, 5 mg/mL; ○, 10 mg/mL.

The growth patterns of* E. coli* incubated with bainiku-ekisu (0–10 mg/mL) are shown in Figure 3. The number of bacteria increased steadily during the 12 hours incubation, reaching approximately 10^8^ CFU/mL when incubated without bainiku-ekisu. The rate of* E. coli *killing was the slowest when using 5 and 10 mg/mL bainiku-ekisu solutions. Under these conditions, the number of bacteria decreased to zero within 24 and 6 hours, respectively. For the 24 hours incubation, the effects of 2.5 mg/mL bainiku-ekisu on* E. coli* were similar to the effects on* S. aureus;* the number of bacteria was approximately 10^1^ CFU/mL. However, the 1 mg/mL bainiku-ekisu solution could kill* E. coli*, and the number of bacteria decreased to approximately 10^2^ CFU/mL. The rate of* E. coli *killing was the fastest for the 1 mg/mL bainiku-ekisu solution among three bacteria. In contrast, the 0.5 mg/mL bainiku-ekisu solution could not inhibit the growth of* E. coli *during the 24 hours incubation. The MIC and MBC of bainiku-ekisu were 1 mg/mL and 2.5 mg/mL, respectively. The MIC and MBC of* Cyperus rotundus* ethyl acetate extract for* E. coli* were 5 and >5 mg/mL, respectively [26]. The antimicrobial activities of bainiku-ekisu against* E. coli* were significantly better than the effects of* C. rotundus* ethyl acetate extract.

Polyphenolic compounds are reported to play a role as antimicrobials [27–29]. Kilani-Jaziri et al. [26] reported that intermediate-polarity polyphenolic compounds (ethyl acetate extract) are active antibacterials. Bainiku-ekisu is a concentrated product that contains full polyphenolic compounds, and therefore, the antimicrobial activity of bainiku-ekisu was outstanding. Kilani et al. [30] reported that coumarin is the major antimicrobial compound in the TOF (Total Oligomers Flavonoids)-enriched extract of* C. rotundus.* Further investigation is required to identify the compound(s) responsible for the antimicrobial activity of bainiku-ekisu.

#### 3.2.2. Growth Rates,* D* Values and* Z* Values

The MICs for* E. faecalis*,* S. aureus,* and* E. coli *were all 1 mg/mL. However, by definition, the real MIC of* E. coli* should be between 0.5 and 1 mg/mL since the numberi of* E. coli* decreased to approximately 10^2^ CFU/mL during incubation with 1 mg/mL. When using this antibiotic, the concentration would be 1 mg/mL, which is slightly high. The MBCs for* S. aureus* and* E. coli *were the same (2.5 mg/mL), but* S. aureus* was more easily destroyed than* E. coli *with 2.5, 5.0 and 10.0 mg/mL bainiku-ekisu (Figures 2 and 3). The exact effective doses might not be the same for* S. aureus* and* E. coli*, thus the growth rate,* D* value and* Z* value were used to calculate MIC and MBC.

**Figure 4:**
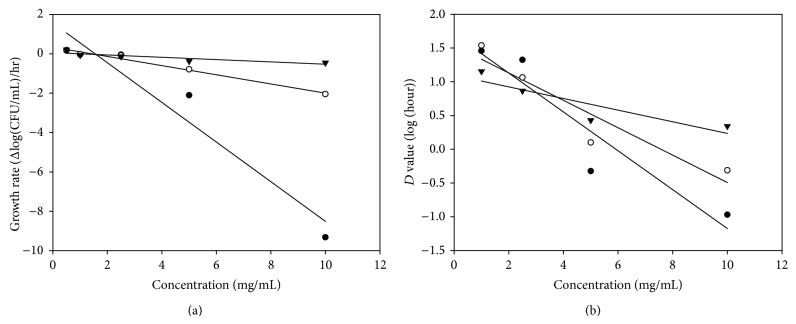
Correlations between the bainiku-ekisu concentration and both the growth rate (a) and the* D* value (decimal reduction time) (b) for* Enterococcus faecalis* (●),* Staphylococcus aureus* (○), and* Escherichia coli* (▼) during incubation.

The linear parts of the growth curves can be expressed with simple linear equations (*R* ^2^ ≥ 0.910), where the slopes were used as the growth rates (*v*_*G*_) (Table 2). The positive growth rate meant that bainiku-ekisu could not kill or inhibit these stains at certain concentration, such as 0.5 mg/mL, and the negative growth rate showed the opposite tendency. MIC was defined that the lowest tested concentration in which the number of bacteria did not vary significantly during 24 hours; that is, the growth rate of the bacterium was around zero. For the MIC calculation, the linear regression of the growth rates with different bainiku-ekisu concentrations is shown in Figure 4(a) and the linear regression equations for* E. faecalis*,* S. aureus,* and* E. coli* were *Y*_1_ = −1.007*X* + 1.556 (*R* ^2^ = 0.9312), *Y*_2_ = −0.234*X* + 0.3335 (*R* ^2^ = 0.9855) and *Y*_3_ = −0.0589*X* + 0.0570 (*R* ^2^ = 0.7767), respectively. *Y*_1_, *Y*_2_, and *Y*_3_ represent the growth rates, and *X* was the bainiku-ekisu concentration. When *Y*_1_, *Y*_2_, and *Y*_3_ set at zero, the calculated MICs (*X*) were found at 1.55, 1.43 and 0.97 mg/mL, respectively. These calculated MICs by growth rates for* E. faecalis, *and* S. aureus* were higher than the MICs by traditional values, and the calculated MIC for* E. coli *showed the opposite result. The MBC was determined to be the lowest tested concentration in which the number of bacteria was reduced 99.9% in CFU/mL; that is, the number of bacteria decreased to 0.1% of the control over 24 hours, and the growth rate of bacterium could be calculated as −0.125 (log⁡10^−3^ CFU/mL/24 hours). When the slope was −0.125 in the regression equation, the MBCs of bainiku-ekisu were 2.59, 2.63 and 2.25 mg/mL for* E. faecalis*,* S. aureus,* and* E. coli*, respectively. These calculated MBCs were different to the traditional MBCs.

The calculated* D* values are listed at Table 2. For the 0.5 mg/mL concentration, the* D* values were not assessable and meaningless because the number of bacteria increased during incubation. A higher* D* value indicates that the bacterium has a higher level of resistance. It is interesting that* E. faecalis *and* S. aureus, *exhibited higher levels of resistance than* E. coli* did at lower bainiku-ekisu concentrations (1.0 and 2.5 mg/mL); in contrast,* E. coli *exhibited a higher level of resistance at higher concentrations (5.0 and 10.0 mg/mL).  Figure 4(b) shows the linear regression of the* D* value versus the bainiku-ekisu concentration. The regression equations for* E. faecalis*,* S. aureus,* and* E. coli* were *Y*_4_ = −0.2878*X* + 1.7036 (*R* ^2^ = 0.8843), *Y*_5_ = −0.2029*X* + 1.536 (*R* ^2^ = 0.8834) and *Y*_6_ = −0.086*X* + 1.0954 (*R* ^2^ = 0.7978), respectively. *Y*_4_, *Y*_5_, and *Y*_6_ were the logarithms of the* D* values, and *X* was the bainiku-ekisu concentration. According to the definition, the* Z* values can be calculated from these equations, and they were 3.47, 4.93 and 11.62 mg/mL for* E. faecalis*,* S. aureus,* and* E. coli*, respectively. The* Z* value of* E. coli* was significantly higher than the values of* E. faecalis,* and* S. aureus*, which indicated that the resistance of* E. coli* against bainiku-ekisu was higher than* E. faecalis,* and* S. aureus. *This result was different to the result from traditional MIC and MBC. Higher resistance of* E. coli* in higher bainiku-ekisu concentrations might exist in some cases, nevertheless, it could not be found in the traditional method.

When the number of bacteria decreased by 99.9% (the definition of MBC), the spent time was equal to 3 times the* D* value for 24 hours, so the* D* value was equal to 8 hours (24 hours/3). Using the regression equation (*Y*_4_, *Y*_5_, and *Y*_6_), the MBCs were determined to be 2.78, 3.12 and 2.24 mg/mL for* E. faecalis*,* S. aureus,* and* E. coli*, respectively. These results are similar to those obtained using the growth rate method. Therefore, the MBC could be calculated from the* D* value but the MIC could not be calculated from* D* value. The difference between calculated values and traditional values might be that the traditional MIC and MBC values were only obtained from the results at 24 hours incubation, but the calculated values were obtained from the results during incubation. The traditional method is easy and fast and the calculated method is exact.

## 4. Conclusion

Bainiku-ekisu could kill or inhibit the growths of* E. faecalis*,* S. aureus,* and* E. coli.* Therefore, bainiku-ekisu could be used as a natural antimicrobial agent to reduce the cross-contamination risk and prevent food poisoning. The MICs and MBCs might be overestimated for pathogens, as higher antibiotic dose to patients might yield antibiotic-resistant bacteria. The determinations of growth rate,* D* value and* Z* value were used to determine the exact cured dose, but these methods are costly and time-consuming. The antibacterial activities of bainiku-ekisu will be tested against other pathogens and further studies will determine the mechanism of antibacterial action of bainiku-ekisu.

## Figures and Tables

**Table 1:** Characteristics of fresh Japanese apricot juice and bainiku-ekisu.

	Fresh juice	Bainiku-ekisu^1^
pH	2.67 ± 0.00^7b8^	2.83 ± 0.02^a^
°Brix	4.9 ± 0.06^a^	4.1 ± 0.03^b^
Total acidity^2^ (g/100 mL)	2.18 ± 0.15^a^	1.77 ± 0.02^b^
Total amount of reducing sugar^3^ (mg/100 mL)	143.11 ± 0.14^a^	55.71 ± 1.34^b^
Total phenolic content^4^ (mg/100 mL)	26.44 ± 0.16^b^	51.00 ± 1.26^a^
Total flavonoid content^5^ (mg/100 mL)	11.39 ± 0.29^b^	16.60 ± 0.8^a^
EC_50_ of DPPH radical-scavenging activity^6^ (mL sample)	0.11 ± 0.00^a^	0.04 ± 0.00^b^
EC_50_ of ABTS^·+^ radical-scavenging activity (mL sample)	0.16 ± 0.00^a^	0.05 ± 0.00^b^

^1^Samples were diluted to the same concentration as fresh juice with de-ionized water.

^2^g citric acid/100 mL sample.

^3^mg glucose/100 mL sample.

^4^mg gallic acid equivalent/100 mL sample.

^5^mg catechin equivalent/100 mL sample.

^6^Volume of sample needed.

^7^Data are presented as the means ± standard deviations (*n* = 3).

^8^Different letters for the individual extracts indicate that the values are significantly different (*P* < 0.05).

**Table 2:** Growth rates and *D* values (decimal reduction time) of *Enterococcus faecalis*, *Staphylococcus aureus,* and *Escherichia coli* treated with various bainiku-ekisu concentrations during incubation.

Bainiku-ekisu concentration (mg/mL)	0.5	1.0	2.5	5.0	10	0.5	1.0	2.5	5.0	10
Strain	Growth rate					*D* value				
	*v*_*G*_ (Δlog⁡(CFU)/hour)					(hour)				
*E. faecalis *	0.162	−0.035	−0.047	−2.106	−9.323	—^1^	28.7	21.1	0.47	0.11
*S. aureus *	0.188	−0.029	−0.086	−0.794	−2.049	—	34.5	11.6	1.26	0.49
*E. coli *	0.199	−0.070	−0.136	−0.372	−0.455	—	14.2	7.34	2.69	2.20

^1^Antibacterial effect is not sufficient to calculate the *D* value.
